# Influence of Dietary Habits on Oxidative Stress Parameters, Selenium, Copper, and Zinc Levels in the Serum of Patients with Age-Related Cataract

**DOI:** 10.3390/nu17203237

**Published:** 2025-10-15

**Authors:** Martyna Falkowska, Izabela Zawadzka, Monika Grabia-Lis, Dominika Patrycja Dobiecka, Maryla Młynarczyk, Joanna Konopińska, Katarzyna Socha

**Affiliations:** 1 Department of Bromatology, Faculty of Pharmacy with the Division of Laboratory Medicine, Medical University of Białystok, Mickiewicza 2D, 15-222 Białystok, Poland; monika.grabia@umb.edu.pl (M.G.-L.);; 2 Department of Ophthalmology, Medical University of Białystok, M. Skłodowskiej-Curie 24a, 15-276 Białystok, Poland

**Keywords:** age-related cataract, oxidative stress, zinc, copper, selenium, diet

## Abstract

Background: A cataract is a clouding of the normally clear lens that obscures the passage of light, effectively reducing clarity and sharpness of vision. Although this disease can affect both children and adults, the most common type is the age-related cataract (ARC). The literature describes many potential agents associated with cataract development. However, this study focuses on modifiable factors, especially nutritional ones and those that may induce oxidative stress. The objective of the present study was to assess serum selenium (Se), copper (Cu), and zinc (Zn) concentrations, as well as the copper/zinc molar ratio (Cu/Zn molar ratio), total antioxidant status (TAS), total oxidant status (TOS), and oxidative stress index (OSI), of patients with ARC in relation to their dietary habits. Methods: A total of 68 patients with ARC and 64 healthy volunteers, with ages ranging from 48 to 92 years, were included in this study. The experimental material collected from the participants consisted of blood samples, which were tested for Se, Cu, and Zn concentrations using atomic absorption spectrometry (AAS). Oxidative stress (OS) parameters, such as TAS and TOS, were estimated spectrophotometrically. In addition, a food frequency questionnaire (FFQ) was used to collect information on the dietary habits of ARC patients. Results: Statistical analysis of the data revealed that the concentrations of Se, Cu, and Zn in serum were significantly lower in ARC patients compared to the controls. In the ARC group, some elements of dietary behavior had a significant effect on the levels of the examined elements and OS parameters. Conclusions: Thus, eventual alterations to one’s diet appear to be worth considering in the context of maintaining homeostasis and adequate mineral levels in ARC patients.

## 1. Introduction

Cataract is a progressive opacification of the naturally transparent lens, which modifies the refraction of light and consequently blurs retinal images [1]. This lens opacity occurs as the crystalline proteins aggregate to form large, water-insoluble complexes that scatter light. Conformational changes in the intracellular lens proteins are grounded in mechanisms of oxidative stress, osmotic pressure changes, and water-protein phase separation [2].

There are two major types of cataract: congenital and acquired. Congenital cataract, also called pediatric cataract, is defined as an opacity of the lens that is present since birth. The primary causes of this condition are genetic or chromosomal mutations, metabolic disorders, related ocular anomalies, and intrauterine infections [3]. It has been established that approximately half of cataract cases in children are due to hereditary factors, some of which are related to systemic abnormalities [4]. In contrast, acquired cataracts develop due to the aging process, an injury, drug-induced effects, or as a manifestation of a systemic disorder. Lens opacities that develop gradually with age without any specific reason are known as senile cataract or age-related cataract (ARC). According to the anatomical location and intensity of lens opacities (Lens Opacities Classification System III) [5], these are divided into nuclear cataract, cortical cataract, and posterior subcapsular cataract (PSC) [4,6]. The estimated prevalence of cataracts in the age-standardized pool indicated nuclear cataracts as the most common of the previously mentioned types [7].

The etiology of the described condition is yet to be fully elucidated. However, oxidative stress, UV exposure, cigarette smoking, alcohol consumption, excessive intake of high-glycemic-index carbohydrates, and malnutrition are reported as the most probable contributing factors. One of the prevailing theories regarding the development of ARC is the oxidative theory, which suggests that free radicals, as highly reactive molecules, cause substantial DNA damage in the epithelial cells of the lens, thereby adversely affecting various cellular components, including proteins, lipids, polysaccharides, and nucleic acids [8,9].

Currently, the most effective and standard method of cataract removal is phacoemulsification. It achieves the best possible visual acuity and optimal postoperative refraction, helping to avoid complications [10]. Although cataract surgery outcomes are generally favorable, this does not exclude the possibility of postoperative sequelae, the most common of which include posterior capsule rupture, transient elevation of intraocular pressure, and posterior capsule opacification. Nevertheless, to this day, surgical methods of cataract removal are the gold standard for managing the condition in question [11].

In an increasingly aware society, attention is being drawn to studies showing links between feasible lifestyle changes and the onset of cataract. A comprehensive dietary approach that includes a balanced diet and vegetarian options, such as fruits, vegetables, legumes, nuts, dairy products, and fish, has been shown to offer protection against cataracts, while a diet high in sodium (Na), fat, and meat may elevate the risk of developing cataracts [12]. Moreover, the association between the occurrence of cataracts and the levels of elements such as zinc (Zn), selenium (Se), calcium (Ca), and sodium is rising in prominence. Nutritional dystrophy, defined as insufficiency of macro- and microelements, is suggested as a possible cause of nuclear cataracts [13].

Statistics show that despite the implementation of new educational programs aimed at preventing vision loss, the reported incidence of preventable blindness and moderate to severe vision impairment (MSVI) increased between 2010 and 2019. According to the studies, in 2020, a total of 15.2 million people over the age of 50 (with a range of 12.7–17.9 million) experienced cataract blindness, while an additional 78.8 million people (with a range of 67.2–91.4 million) were affected by MSVI. These findings place cataract as the leading and the second-leading cause of blindness and MSVI in those aged 50 years and older, respectively [14].

Hence, considering both the magnitude of the problem and the potential for mitigation, it is crucial to explore new approaches to cataract prevention. Thus, the present study focuses on modifiable risk factors for cataract occurrence with particular attention to the relationship between dietary habits and redox balance among ARC patients.

## 2. Materials and Methods

### 2.1. Characteristic of the Study Groups

The study group comprised 68 patients (aged 48–92 years) with ARC from the Department of Ophthalmology, Medical University of Białystok Clinical Hospital (Poland) and 64 healthy individuals (aged 49–83 years) recruited from the patients’ family members, hospital personnel, and university employees who volunteered at the Bromatology Department of the Medical University of Białystok (MUB).

The health status of patients from the ARC group and healthy volunteers in the control group was evaluated by an independent ophthalmology specialist at the Department of Ophthalmology of the Medical University of Białystok Clinical Hospital (Poland). To diagnose patients in the study group and exclude eye diseases in the control group, participants underwent examination of the anterior segment of the eye and the ocular fundus, and had their intraocular pressure measured. The above-mentioned ophthalmological examinations utilized a slit lamp, recognized as the ideal tool for evaluating cataracts, as it provides information on the anatomical location of lens opacities. Cataract removal surgery was recommended if vision loss significantly limited the patient’s daily functioning or the ability to perform professional duties, and improvement in visual acuity was impossible without surgical intervention. Moreover, the standards published by the Polish Ophthalmological Society recommend qualification for cataract surgery for patients whose visual acuity is 0.6 or less at a distance, except in urgent cases [15]. Patients diagnosed with ARC, before surgical removal, underwent standard diagnostic tests at the referenced hospital, which included peripheral blood smear (PBS), fasting blood sugar (FBS), liver function tests (LFTs), blood pressure (BP), heart rate (HR), and electrocardiography (ECG). Any irregularities in those parameters resulted in the patients’ temporary disqualification from phacoemulsification surgery. According to the protocol, this was the case until the health status of the ARC patients normalized, which was facilitated by cooperation with the general practitioner. Patients in the ARC group were those who were eligible for cataract extraction surgery and met the criteria described above.

Exclusion criteria for joining the study included comorbidities, such as systemic diseases, especially liver and intestinal disorders resulting in malabsorption syndrome, hyperthyroidism, hypovitaminosis, psychiatric disorders, autoimmune diseases, and neoplasms. In addition, patients with a history of alcohol abuse syndrome, ophthalmological surgery, ocular inflammation, retinal vascular diseases, or diabetic retinopathy were excluded from the study. Furthermore, individuals taking dietary supplements containing minerals, particularly those with Se, Cu, and Zn, were also excluded.

The modified food-frequency questionnaires (FFQ) developed by the Committee of Human Nutrition Science, Polish Academy of Sciences, were employed to collect the dietary data. Patients with ARC were asked to complete a questionnaire concerning the frequency of consumption of different food products. The list consisted of 36 groups of food items (bacon, beer, boiled vegetables, butter, coffee, cottage cheese, eggs, farinaceous dishes, fresh fish, fruit, grain products, ham, honey, jam, margarine, cured meat, meat, milk, offal, oils, other kinds of cheese, potatoes, poultry, pulses, raw vegetables, sausages, soft drinks, sugar added to beverages, sweets, tea, tinned fish, tinned meat, vodka, white bread, whole-grain bread, and wine). Frequent consumption was defined as having specific products at least 12 times a month, with the exception of fish, for which 4 times a month was considered a frequent consumption pattern. Less frequent consumption of specific foods was termed “occasional consumption.” The nutritional status of patients was assessed using basic anthropometric measurements, including self-reported height, weight, and BMI. Each patient completed a questionnaire that included general information, medical history, and dietary habits. Additionally, a 3-day dietary interview was conducted to estimate accurate nutrient intake. The data were compared with Polish Nutrition Standards [16]. The study protocol was approved by the Bioethics Committee of the MUB (APK.002.137.2022; Białystok, 24 March 2022). All participants provided written consent to participate in the study.

### 2.2. Collection and Preparation of Samples

The experimental materials consisted of blood (approximately 6 mL) from the study participants, which was collected using S-Monovette tubes with clotting activator, suitable for elemental analysis (S-Monovette K3E: 1.6 mg EDTA/mL vacuum blood collection tube; Sarstedt AG & Co., Nümbrecht, Germany). The samples were centrifuged for 10 min at approximately 2500 rpm to collect serum, which was then stored at −20 °C. Serum Se concentration was ascertained directly after diluting 1:1 with 0.2% Triton X-100. In advance of determining serum Zn and Cu concentrations, the serum samples were deproteinated using 1 mol/L spectral-grade nitric acid (Merck, Darmstadt, Germany). A 1% Triton X-100 solution was then added, mixed using a vortex, and centrifuged for 10 min. The concentration of Zn was quantified in the supernatant, and the content of Cu was established after dilution in 0.1 mol/L nitric acid.

### 2.3. Determination of Mineral Components

Serum concentrations were assayed via electrothermal atomic absorption spectrometry (for Se and Cu) and flame atomic absorption spectrometry (for Zn). These measurements utilized Zeeman background correction at respective wavelengths of 196 nm, 324.8 nm, and 213.9 nm (Z2000 instrument, Hitachi, Tokyo, Japan). Working solutions were prepared from standard solutions at a concentration of 1 g/L for curve calibration (Merck, Darmstadt, Germany). The analytical detection limits were determined at 1.88 µg/L for Se, 0.52 µg/L for Cu, and 0.014 mg/L for Zn.

To evaluate the accuracy and precision of the analytical techniques, certified human serum reference material (Seronorm Trace Elements, Serum Level 1, 0903106, Sero AS, Billingstad, Norway) was employed. Every result obtained from the control samples matched the corresponding reference values. The methods demonstrated a precision of 3.3% for Se, 2.3% for Cu, and 1.9% for Zn determination.

The measured concentrations of Se, Cu, and Zn in the serum were interpreted in relation to the reference values: 66–104 µg/L, 0.7–1.6 mg/L, and 0.7–1.3 mg/L, respectively [17]. Microsoft Excel was used to calculate the Cu/Zn molar ratio. The limits for the Cu/Zn ratio were in a range of 0.6–1.0.

### 2.4. Determination of Total Antioxidant Status and Total Oxidant Status

Serum total antioxidant status (TAS) was determined spectrophotometrically at a 600 nm wavelength, employing commercially available test kits from Randox Laboratories Ltd. (Crumlin, UK) and a spectrophotometer (Epoch, Agilent, Glostrup, Denmark). The process involved incubating ABTS^®^ (2,20-Azino-di-[3-ethylbenzthiazoline sulphonate]) with peroxidase (metmyoglobin) and H_2_O_2_ to generate the ABTS^®^* + radical cation, which produces a relatively stable blue-green hue detectable at 600 nm. Antioxidants present in the added samples decreased this color formation in direct proportion to their concentrations. Method accuracy was confirmed using the TAS Control kit (Randox Laboratories Ltd., Crumlin, UK), with the serum TAS reference range established as 1.3–1.77 mmol/L [18].

Total oxidant status (TOS) was assessed using a spectrophotometric approach via a microplate reader (Epoch, Agilent, Glostrup, Denmark), in accordance with the method described by Erel et al. [19] at wavelengths of 560 nm and 800 nm (Table 1). The oxidative stress index (OSI) was calculated as the TOS/TAS ratio.

For assigning results as either low or high, a normal reference range was established for each parameter, encompassing acceptable normal values and accounting for the margin of error. The set limits were 1.45–2.00 mmol/L for TAS, 5–8 μmol H_2_O_2_ Equiv./L for TOS, and 0.3–0.6 for OSI.

### 2.5. Statistical Analysis

Statistical analyses were conducted using Statistica v.13.0 (TIBCO Software Inc., Palo Alto, CA, USA). The normal distribution of the data was determined using the Kolmogorov–Smirnov and Shapiro–Wilk tests. Differences between independent groups were examined using the Mann–Whitney U test or the Kruskal–Wallis ANOVA. For evaluating the correlation between qualitative variables, Pearson’s chi-square test was applied. Spearman’s correlation coefficient was employed to assess the strength and direction of the monotonic correlation between two variables. A stepwise multiple linear regression analysis was conducted to estimate the influence of dietary habits on serum Se, Cu, Zn, and TAS, TOS, and OSI levels in the study group. Differences at a *p* < 0.05 significance level were considered statistically significant.

## 3. Results

The results were analyzed based on data from both cataract patients in the ARC group (*n* = 68) and healthy volunteers in the control group (*n* = 64). Table 2 presents characteristics of the study participants.

Of all the study participants, 63% of the ARC group and 83% of the control group were women, while men comprised 37% and 17% of the aforementioned groups, respectively.

The study found statistically significant differences between the ARC group and the control group in serum concentrations of Se, Cu, and Zn (*p* < 0.001, *p* < 0.005, and *p* < 0.001, respectively). The tested elements were significantly lower in the cataract patients compared to healthy controls, with median levels of Me_Se_ = 55.22 µg/L, Me_Cu_ = 0.869 mg/L, Me_Zn_ = 0.755 mg/L, Me_Se_ = 73.90 µg/L, Me_Cu_ = 0.993 mg/L, and Me_Zn_ = 0.856 mg/L, respectively. TAS, TOS, OSI, and Cu/Zn ratio revealed no statistically significant difference between the groups; however, there was a positive correlation between the presence of the disease and those parameters (r_TAS_ = 0.0732, r_TOS_ = 0.1468, r_OSI_ = 0.0680, r_Cu/Zn_ = 0.0167, respectively). The comparison between genders revealed that women had a lower serum Se concentration than men. Additionally, female patients from the ARC group had statistically significantly lower serum concentrations of Se, Cu, and Zn compared to healthy female controls. Similarly, male patients from the ARC group had lower blood levels of Se and Zn than healthy male controls. The results are summarized in Table 3.

Although there was no statistically significant difference in the concentration of the examined parameters depending on the body mass index (BMI) or age of study participants, the serum Zn concentrations appeared to be statistically significantly correlated with the smoking status of patients with ARC (r = −0.2416, *p* < 0.05).

Significant differences in the proportion of participants with low, normal, or high serum levels of Se, Cu, Zn, TAS, and TOS in comparison to the reference values were observed between the ARC and control groups (*p* < 0.001, *p* < 0.005, *p* < 0.005, *p* < 0.05, *p* < 0.05, respectively). The proportions of participants with normal levels of Se, Cu, Zn, TAS, and TOS were 25%, 59%, 62%, 53%, and 50% in the ARC group, and 52%, 86%, 81%, 41%, and 47% in the control group, respectively. A higher percentage of participants in the ARC group than controls had serum concentrations of Se, Cu, and Zn below the standard value (75% vs. 31% for Se; 38% vs. 11% for Cu; 38% vs. 13% for Zn), while for TAS, it was identical in both groups at 47%. Unlike the previously mentioned parameters, 46% of participants in the ARC group and 34% in the control group had TOS levels below the standard value. Moreover, approximately 17%, 6%, and 19% of the controls had serum levels of Se, Zn, and TOS that were higher than the reference range for those elements. The number of participants with Cu concentrations above the reference range was around 3% for both the ARC and control groups. About 12% of the controls had very good serum TAS levels. The comparison of Cu/Zn molar ratio and OSI in relation to their reference range among patients with ARC and controls remained statistically insignificant. The results are presented in Table 4.

Stepwise multiple linear regression analysis revealed that selected dietary habits may positively or negatively impact the concentrations of micronutrients and OS parameters in the serum of patients with ARC (Table 5). Of the 36 food product groups listed in the Methods Section, 28 were included in the analysis. Due to the rare consumption of offal, bacon, tinned meat, sweet drinks, beer, wine, and vodka, as well as the frequent consumption of fruit by a significant proportion of the population (at least 90%), these were excluded from the regression model presented in the study. The low variability in the frequency of consumption of the eight variables implied considering them as non-influential factors. Frequent consumption of full-fat cheese was statistically significantly correlated with increased serum Se concentrations in ARC patients, whereas frequent consumption of potatoes and butter was statistically significantly associated with lower serum Se levels. For Cu, a significant positive relationship was noted for milk consumption, and a negative one for margarine and oil intake. In addition, significant positive correlations were observed between Zn concentrations and the consumption of white bread, whole-grain bread, legumes, fish, cured meat, and coffee. In contrast, high intake of butter and potatoes was connected with lower serum Zn levels. For the Cu/Zn ratio, a significant positive correlation was observed with milk consumption, and a negative one with oil ingestion. In case of TAS, TOS, and OSI, there was a statistically significant relationship between tea and groats and rice intake and TAS levels, margarine, fish, milk, tea, boiled vegetables, and potatoes ingestion and TOS, as well as groats and rice, margarine, tea, milk, boiled vegetables, potatoes, and coffee and OSI. The independent variables included in the model accounted for about 25%, 14%, 36%, 17%, 25%, 33% and 33% of the variance for Se, Zn, Cu, Cu/Zn molar ratio, as well as TAS, TOS, and OSI, respectively.

Dietary intake of vitamins, micro- and macronutrients, as well as the percentages of ARC patients fulfilling the dietary nutrient norms, are presented in Table 6. Analysis of the collected data revealed deficiencies in patients’ diets in terms of recommended intake of potassium, calcium, magnesium, folic acid, vitamin D, omega-3 fatty acids, and dietary fiber. The highest percentage of patients with nutrient intake below the recommended norm was observed for vitamin D (F = 100%, M = 100%), calcium, folic acid (F = 95%, M = 80%), omega-3 fatty acids (F = 86%, M = 76%), and potassium (F = 53%, M = 72%). Furthermore, excessive consumption of sodium (Me = 3269.788 mg/day (2637.895–4008.582)), phosphorus (Me = 1130.194 mg/day (855.115–1406.868)), and sodium chloride (Me = 8.179 g/day (6.599–9.851)) was observed among the participants in the study group, as well as an increase in the percentage of energy obtained from fat (Me = 33.255% E (30.775; 36.825)) and carbohydrates (Me = 46.650% E (43.525–50.125)) in their daily diet. A comparison of the median intake of specific nutrients by women and men revealed statistically significant differences in the intake of sodium (Me_F_ = 3072.69 mg/day, Me_M_ = 3694.83 mg/day, *p* < 0.005), retinol (Me_F_ = 848.81 µg/day, Me_M_ = 1142.82 µg/day, *p* < 0.05), thiamine (Me_F_ = 1.06 mg/day, Me_M_ = 1.27 mg/day, *p* < 0.005), salt (Me_F_ = 7.68 g/day, Me_M_ = 9.24 g/day, *p* < 0.005), fat (Me_F_ = 32.51%, Me_M_ = 34.50%, *p* < 0.05), and carbohydrates (Me_F_ = 47.72%, Me_M_ = 45.54%, *p* < 0.05). There was a statistically significant correlation between vitamin A and β-carotene consumption and TOS (r = −0.251219, r = −0.259426, respectively).

Table 7 and Figures S1–S6 present statistically significant associations between blood serum parameters and dietary or lifestyle choices among ARC patients. Statistically significant negative correlations were found between the Cu/Zn molar ratio and dietary Zn, Cu, and Mn intake, TOS level, and vitamin A and β-carotene consumption, as well as serum Zn concentration and patients’ smoking status.

## 4. Discussion

Oxidative stress (OS), resulting from redox imbalance, plays a pivotal role in many ocular degenerative disorders, including ARC. The principal factors contributing to lens oxidative damage are reactive oxygen species (ROS). An excess of those oxygen-derived molecules, due to their high reactivity, can affect several intracellular components, especially proteins, lipids, and nucleic acids [20,21]. Consequently, by competing for paired electrons, ROS can trigger lipid peroxidation, protein modification, and induce defects of chromosomes and mitochondrial DNA (mtDNA). Hence, the aforementioned oxidants may alter information transfer and gene expression, which eventually promote autophagy, apoptosis, and necrosis [22].

To shield against oxidative stress damage, organisms employ antioxidant defense systems, which contain antioxidants to prevent or slow down oxidation processes. The cellular systems in most cell types are divided into non-enzymatic and enzymatic ones and account for the removal of free radicals and non-radical oxidants [23]. The lens of the eye is equipped with several enzymes that collectively neutralize superoxide anion radicals (O_2_•^−^), which are superoxide dismutase (SOD), catalase (CAT), and glutathione peroxidase (GPX). In addition to enzymatic defense systems, the lens also contains non-enzymatic antioxidants, among which the primary are reduced glutathione (GSH), vitamin C, and vitamin E. Furthermore, key trace elements, such as Se, Cu, and Zn, are essential for the proper performance of the above-mentioned defense systems [20,24].

Se is a micronutrient involved in numerous processes, among which the most important in cataract formation is selenoprotein synthesis. Particularly essential selenoproteins are GPX and thioredoxin reductase (TrxR) [25], which, by neutralizing peroxides, promote antioxidant defense [20]. GPX activity is determined by Se availability, whose suggested serum concentration in humans, optimal for GPX and other selenoprotein functions, ranges from 66 to 104 µg/L [17]. In this study, a significantly lower concentration of Se was observed in the ARC group in comparison to the control group (Me_Se_ = 55.22 µg/L and Me_Se_ = 73.90 µg/L, respectively). Moreover, analysis of Se levels in relation to its reference range revealed significant disruption, highlighted especially in the group of patients with ARC (below norm: 75% of the ARC group, 31% of the control group). The findings of Post et al. also suggest that the prevalence of ARC may be secondary to the decreased serum Se concentrations (OR_nuclear cataract_ = 12.82, *p* < 0.01; OR_cortical cataract_ = 3.31, *p* < 0.01). Altogether, the researchers remarked that an understanding of serum Se levels in ARC patients can provide important insights into lens metabolism and thus become an indicator of its alterations [26].

Cu and Zn are trace elements that, as enzyme cofactors, play a pivotal role in neutralizing superoxide anions by stimulating Cu/Zn–SOD activity. Recommended intake ranges for adults are 0.7–1.6 mg/L for serum Cu and 0.7–1.3 mg/L for serum Zn concentrations [17]. Levels of the tested elements in the present study were statistically significantly lower among ARC patients than healthy controls, with median levels of Me_Cu_ = 0.87 mg/L, Me_Zn_ = 0.76 mg/L, Me_Cu_ = 0.99 mg/L, and Me_Zn_ = 0.86 mg/L, respectively. Similarly to Se, statistically significant differences were observed between the ARC group and the control group in terms of meeting the reference range for the concentrations of Cu and Zn (below norm: 38% and 38% of the ARC group, 11% and 13% of the control group, respectively). The results of Chakrabordy et al., like the current study, showed significantly lower serum Zn (M_cataract group_ = 0.64 mg/L, M_control group_ = 0.97 mg/L, *p* < 0.05) and SOD concentrations in the cataract group in comparison to the controls (M_cataract group_ = 3.28 U/mL, M_control group_ = 4.06 U/mL, *p* < 0.001) [27]. Additionally, while in the study by Akyol et al., among patients with cataracts, serum Zn concentrations were within the reference range (M_Zn_ = 1.37 mg/L, reference range indicated by the authors: 0.8–1.4 mg/L), slightly higher values were observed in relation to the serum Cu concentrations (M_Cu_ = 1.85 mg/L; reference range indicated by Akyol et al.: 0.9–1.6 mg/L). The observation presented in the study was explained by disease-related stress, with Cu levels increasing and Zn levels decreasing, which is associated with the acute phase response [28].

In this particular study, the medians of OS components did not indicate statistically significant differences between the ARC group (Me_TAS_ = 1.45 mmol/L, Me_TOS_ = 5.49 µmol H_2_O_2_ equiv./L, Me_OSI_ = 0.35) and the control group (Me_TAS_ = 1.51 mmol/L, Me_TOS_ = 5.75 µmol H_2_O_2_ equiv./L, Me_OSI_ = 0.38). However, the comparison of serum TAS and TOS among patients with ARC and the control groups in relation to their reference range revealed statistically significant differences. Upon searching the literature for references, the authors identified a gap in research on TAS, TOS, and OSI in the blood serum of patients with ARC. Nevertheless, a review by Hsueh et al. [22] found that the total antioxidant capacity (TAC) of blood serum [29,30] and eye lenses [29,31] in patients with cataracts, compared to the control group, appeared to be lower among individuals with ARC. Patients with cataracts also had lower levels of vitamin E, vitamin C [29,32], and β-carotene [33], which were associated with an increased risk of ARC. However, said correlation was not observed in the analysis of the diets of the patients included in our study (Me_Vitamin E_ = 9.27 mg/day, Me_Vitamin C_ = 83.18 mg/day, Me_β-carotene_ = 3677.59 µg/day).

Various nutritional factors can influence mineral concentrations in the human body, either directly by being a source of the mineral or indirectly by affecting its dietary bioavailability positively or negatively [34]. The current study suggests that certain dietary modifications, specifically the selection and frequency of specific food intake, may enhance the status of the antioxidant elements investigated in the ARC group. For instance, stepwise multiple linear regression analysis of the influence of the frequency of food product ingestion on the levels of certain elements and redox balance status in the serum of patients with ARC has shown that reducing the frequency of potato, butter, oil, margarine, and poultry consumption could be beneficial. On the other hand, frequent consumption of full-fat cheese, milk, white and whole-grain bread, legumes, fish, cured meat, and coffee appeared to improve the mutual proportions between Se, Cu, and Zn. Furthermore, beneficial effects on the antioxidant status could also be achieved by altering the intake of tea, groats, rice, and boiled vegetables.

The links between diet and ARC have been widely described in the literature. However, many of these reports are based on individual studies, which, due to their lack of reproducibility, cannot be used to establish uniform dietary recommendations that would form the dietary baseline for preventing or slowing the progression of ARC. In the presented publication, an analysis of 3-day food diaries of ARC patients revealed considerable, i.e., affecting at least half of the study population, and statistically significant deficiencies in the intake of K, Ca, Mg, folic acid, vitamin D, omega-3 unsaturated fatty acids, and dietary fiber, as well as statistically significant excessive consumption of Na, salt, fat, and carbohydrates. To determine dietary errors as accurately as possible, the results obtained from the Diet 6.0 program were compared with the latest nutritional standards established for the Polish population [16].

Comparing the results with data from the literature, an ion imbalance caused by an increase in Ca and Na concentration and a decrease in K concentration in the lens of the human eye affected by senile cataract was found [35]. Furthermore, lower concentrations of K have been observed in both the plasma [36] and the lens [37] of patients with cataracts. Research indicates that either an excess or a deficiency of Ca can contribute to the development of cataracts. In fact, strongly localized opacities were found in lenses with the highest Ca concentration and almost normal Na levels, whereas low Ca content was observed in those with nuclear cataracts. As the authors suggest, Ca deficiency in patients with ARC may be associated with osteoporosis, as this disease is one of the known risk factors for cataracts [38]. The effect of Mg deficiency on the development of lens opacity has been described as multifactorial. It includes cell membrane dysfunction caused by adenosine triphosphate (ATP) depletion, excessive nitric oxide (NO) production, which predisposes to ion imbalance by altering gap junction proteins, oxidative stress leading to lens fiber apoptosis, and further contributing to cataract formation [35,39]. Data on the role of folic acid in eye health are inconclusive. The data collected in Taiwan in the Elderly Nutrition and Health Survey indicate that, compared to men with folic acid deficiency, individuals with adequate folic acid levels had a lower risk of developing “any” type of cataract (OR = 1.5, 95% CI [1.02–2.29]). This effect was evident in men over 75 (OR = 3.4, 95% CI [1.52, 7.53]) as well as in those with adequate vitamin B2, B6, and B12 status (OR = 2.6, 95% CI [1.30, 5.37]) [40]. However, the European Prospective Investigation into Cancer and Nutrition (EPIC-Oxford) study found no statistically significant association between the risk of developing “any” type of cataract and folic acid intake (IRR = 1.01, 95% CI [0.83, 1.22], *p* < 0.559) [41]. A meta-analysis from 2025 revealed a correlation between serum vitamin D concentrations and cataract. Lower serum vitamin D levels were associated with a greater prevalence of cataract (*p* = 0.047, MD: −4.87, 95% CI [−9.67, −0.07]). Furthermore, this negative correlation was observed in both male (*p* = 0.01, MD: −2.15, 95% CI [−3.83, −0.46]) and female patients (*p* < 0.01, MD: −6.67, 95% CI [−8.20, −5.14]). Additionally, analysis among the population with different types of cataracts showed a significant association between serum vitamin D level and nuclear (*p* < 0.01, MD: −10.48, 95% CI [−12.72, −8.24]) and PSC (*p* = 0.02, MD: −6.05, 95% CI [−11.30, −0.80]) but not in cortical cataract (*p* = 0.14, MD: −6.74, 95% CI [−15.70, 2.22]) [42]. The omega-3 dietary pattern, characterized by increased DHA and EPA content, was statistically significantly associated with a lower risk of ARC (OR = 0.71, 95% CI [0.40–0.92], *p* < 0.05) [43]. The correlation between fiber-rich products, such as fruits and vegetables, and a reduced risk of ARC has been demonstrated in numerous studies conducted worldwide [44,45,46,47,48].

Concerning excessive consumption of specific nutrients and increased risk of ARC, salt and carbohydrates are most frequently mentioned, much like in the present study. The Blue Mountains Eye Study found an association between high dietary Na intake and PSC and suggested that a low-sodium diet may help prevent the development of cataracts in elderly populations [49]. The result of meta-analysis revealed a significant correlation between higher carbohydrate ingestion and risk of cortical cataract (OR = 1.37, 95% CI [0.99–1.90]) and a statistically significant association between higher glycemic index and risk of nuclear cataract (OR = 1.23, 95% CI [1.03–1.46]) [50].

The links between serum parameters and dietary or lifestyle choices of ARC patients were found to be statistically significant for a limited set of six associations. TOS was negatively correlated with vitamin A and β-carotene intake, Zn concentration with cigarette smoking, and Cu/Zn molar ratios with Zn, Cu, and Mn ingestion. The predominant sources of vitamin A are known to be animal-derived products, such as liver, butter, eggs, full-fat dairy products, and certain species of fish. Plant-based products, especially brightly colored vegetables and fruits like carrots, spinach, red peppers, and pumpkin, mainly contain their provitamin, β-carotene. Those compounds are responsive to OS by either altering their impact on gene expression or delaying lipid oxidation-induced radical propagation [51]. Furthermore, they may exhibit antioxidant activity and, as confirmed by the present studies, affect TOS in the blood. The negative impact of smoking in ARC patients on serum Zn concentration was proven as early as 2009, when Al-Timimi et al. noticed an increased incidence of marginal hypozincemia among smokers compared to non-smokers [52]. Additionally, the influence of dietary intake of Mn, Zn, or Cu on Cu/Zn molar ratios, whose complex, often competitive interactions affect their respective levels in the body, was observed by Grabia et al. [53].

The present study has some limitations and strengths that should be considered. Its notable strength lies in the fact that it fills the literature gaps regarding the correlations between serum trace elements, OS parameters, and dietary habits in the elderly with ARC to spread awareness about their potential links. The other advantages of this research include a moderate-sized sample, a clinical-based, single-center design, and standard documentation of cataracts using the LOCS III. All of this implies that collected data have the potential to be useful in developing long-term strategies to combat treatable blindness. The main limitation of this study is its retrospective nature and the insufficient validation of the causal relationship between risk factors, such as sunlight exposure, and cataracts. Furthermore, responses provided in self-administered questionnaires might be subject to bias. The problem of overestimating or underestimating the amount and frequency of food consumption seems to be common, especially among elderly patients. Therefore, in order to improve the quality of future studies, it would be advisable to expand self-reported survey forms with control questions to verify the reliability of data obtained from patients. As an alternative to the above solution, the assistance of a dietitian should be considered, who, as a qualified specialist, could work with patients in the ward to collect dietary histories or provide instructions on completing nutrition questionnaires correctly. Moreover, when selecting individuals for the control group, particular attention was paid to their health status. By prioritizing this aspect, the authors were compelled to compromise on the age of healthy volunteers. Additionally, since the healthy volunteers in the control group refused to participate in the survey, the anthropometric and nutritional data for this population were not included in the present study. Hence, data on diet, as well as information on BMI and systemic factors known to influence ARC, such as diabetes, hypertension, corticosteroid use, and smoking status, were lacking in healthy individuals. Thus, the inability to assess the above-mentioned factors between the ARC group and the control group is a limitation of the presented research, and caution should be exercised in interpreting the study results.

## 5. Conclusions

According to recent studies, it can be stated that a variety of dietary mistakes, leading to deficiencies in crucial antioxidant components, can interfere with the body’s redox balance. Consequently, by affecting oxidative stress, these could serve as a possible contributor to ARC. Therefore, ensuring stability and proper mineral intake through dietary modifications seems essential for individuals with ARC.

## Figures and Tables

**Table 1:** Detailed specifications for the elements, antioxidant defense markers, and oxidative stress markers measured.

Parameter	Unit	Wavelength	Material
Se	µg/L	196 nm	Serum
Cu	mg/L	324.8 nm	Serum
Zn	mg/L	213.9 nm	Serum
TAS	mmol/L	600 nm	Serum
TOS	μmol H_2_O_2_ equiv./L	560/800 nm	Serum

Abbreviations: Se—selenium, Cu—copper, Zn—zinc, TAS—total antioxidant status, TOS—total oxidant status.

**Table 2:** Characteristics of the study groups.

	ARC Group (*n* = 68)	Control Group (*n* = 64)
Gender (F/M)	43/25	53/11
Age (years): median (min.–max.)	72 (48–92)	57 (49–83)
Height (m): median (min.–max.)	1.65 (1.4–1.82)	-
Weight (kg): median (min.–max.)	75 (50–118)	-
BMI (kg/m^2^): median (min.–max.)	28.04 (17.28–48.89)	-
Smoking status: smoker (*n*)/non-smoker (*n*)	21/47	-
Alcohol intake: drinker (*n*)/ non-drinker (*n*)	31/37	-

Abbreviations: ARC—age-related cataract, F—female, M—male.

**Table 3:** Concentrations of selenium, copper, zinc, copper/zinc molar ratio, total antioxidant status, total oxidant status, and oxidative stress index in the serum of patients with ARC and healthy controls.

	ARC Group		Control Group		*p*-Value
	Median (Q1–Q3)				
	F (a) (*n* = 43)	M (b) (*n* = 25)	F (c) (*n* = 53)	M (d) (*n* = 11)	
Se	55.22 (46.66; 66.03)		74.24 (62.34–90.77)		<0.001
	57.34 (49.30–66.16)	54.25 (40.10–65.90)	73.60 (62.79–90.41)	79.99 (61.23–91.13)	a vs. c * b vs. d * ac vs. bd *
Cu	0.869 (0.583–1.101)		0.995 (0.817–1.145)		<0.005
	0.873 (0.605–1.124)	0.864 (0.566–0.990)	0.993 (0.807–1.189)	1.017 (0.858–1.108)	a vs. c *
Zn	0.755 (0.645–0.828)		0.855 (0.745–0.958)		<0.001
	0.766 (0.660–0.838)	0.735 (0.634–0.778)	0.853 (0.745–0.954)	0.901 (0.739–0.961)	a vs. c * b vs. d *
Cu/Zn	1.141 (0.810–1.514)		1.168 (0.941–1.352)		ns
	1.154 (0.798–1.520)	1.134 (0.823–1.402)	1.163 (0.949–1.358)	1.188 (0.780–1.346)	-
TAS	1.452 (1.276–1.667)		1.5117 (1.2195–1.7753)		ns
	1.452 (1.307–1.667)	1.429 (1.241–1.684)	1.570 (1.224–1.747)	1.396 (1.062–2.309)	-
TOS	5.494 (4.217–6.228)		5.753 (4.655–7.327)		ns
	5.666 (4.247–6.315)	4.600 (4.135–5.892)	5.722 (4.623–7.150)	6.191 (4.784–9.406)	-
OSI	0.354 (0.268–0.431)		0.3775 (0.2537–0.5852)		ns
	0.367 (0.268–0.425)	0.320 (0.268–0.437)	0.375 (0.258–0.555)	0.428 (0.215–0.811)	-

Statistically significant differences between the levels were detected using the Mann–Whitney U test analysis. Abbreviations: ARC—age-related cataract, Se—selenium, Cu—copper, Zn—zinc, Cu/Zn molar ratio—copper/zinc molar ratio, TAS—total antioxidant status, TOS—total oxidant status, OSI—oxidative stress index, Q1—lower quartile, Q3—upper quartile, *p*—level of significance, ns—not significant, a—female from ARC group, b—male from ARC group, c—female control, d—male control, * *p*-value < 0.05.

**Table 4:** The comparison of serum selenium, copper, zinc, copper/zinc molar ratio, total antioxidant status, total oxidant status, and oxidative stress index levels among patients with ARC and healthy controls in relation to the reference range.

Reference Range	Group Classification	Study Group		*p*-Value (ARC Patients vs. Controls)
		ARC Group (*n* = 68)	Control Group (*n* = 64)	
Se (selenium)				
66–104 µg/L	Low: *n* (%)	51 (75%)	20 (31%)	<0.001
	Normal: *n* (%)	17 (25%)	33 (52%)	
	High: *n* (%)	0 (0%)	11 (17%)	
Cu (copper)				
0.7–1.6 mg/L	Low: *n* (%)	26 (38%)	7 (11%)	* <0.005
	Normal: *n* (%)	40 (59%)	55 (86%)	
	High: *n* (%)	2 (3%)	2 (3%)	
Zn (zinc)				
0.7–1.3 mg/L	Low: *n* (%)	26 (38%)	8 (13%)	* <0.005
	Normal: *n* (%)	42 (62%)	52 (81%)	
	High: *n* (%)	0 (0%)	4 (6%)	
Cu/Zn (Cu/Zn molar ratio)				
0.6–1.0	Low: *n* (%)	10 (15%)	2 (3%)	ns
	Normal: *n* (%)	15 (22%)	18 (28%)	
	High: *n* (%)	43 (63%)	44 (69%)	
TAS (total antioxidant status)				
1.45–2.0 mmol/L	Low: *n* (%)	32 (47%)	30 (47%)	* <0.05
	Normal: *n* (%)	36 (53%)	26 (41%)	
	High: *n* (%)	0 (0%)	8 (12%)	
TOS (total oxidant status)				
5–8 μmol H_2_O_2_ Equiv./L	Low: *n* (%)	31 (46%)	22 (34%)	<0.05
	Normal: *n* (%)	34 (50%)	30 (47%)	
	High: *n* (%)	3 (4%)	12 (19%)	
OSI (oxidative stress index)				
0.3–0.6	Low: *n* (%)	21 (31%)	22 (34%)	ns
	Normal: *n* (%)	39 (57%)	27 (42%)	
	High: *n* (%)	8 (12%)	15 (24%)	

Statistically significant differences between the ranges were detected using Pearson’s chi-square test with Yates’ continuity correction analysis. Abbreviations: ARC—age-related cataract, *p*—level of significance, ns—not significant, *—Yates’ *p*-Value.

**Table 5:** Stepwise multiple linear regression analysis of the influence of food products on the levels of total antioxidant status, total oxidant status, oxidative stress index, selenium, copper, zinc, and copper/zinc molar ratio in the serum of patients with ARC.

Independent Variables	β Coefficient	SE	*p*-Value	Adj. R^2^
Se (selenium)				
Full-fat cheese	0.252	0.117	0.0359	0.25
Oils	0.258	0.130	0.0522	
Sausages	0.204	0.129	0.1215	
Groats and rice	0.179	0.122	0.1471	
Milk	0.164	0.127	0.2043	
Sweetbread	0.161	0.131	0.2271	
Potatoes	−0.381	0.128	0.0043	
Butter	−0.260	0.126	0.0444	
Farinaceous dishes	−0.179	0.113	0.1206	
Tinned fish	−0.181	0.115	0.1218	
Jam	−0.158	0.114	0.1724	
Margarine	−0.167	0.127	0.1938	
Tea	−0.145	0.114	0.2091	
Raw vegetables	−0.153	0.126	0.2312	
Cu (copper)				
Milk	0.342	0.123	0.0069	0.14
Sugar	0.177	0.121	0.1506	
Oils	−0.280	0.120	0.0233	
Margarine	−0.270	0.117	0.0238	
Potatoes	−0.146	0.118	0.2196	
Zn (zinc)				
White bread	0.411	0.126	0.0019	0.36
Whole-grain bread	0.305	0.115	0.0104	
Legumes	0.280	0.106	0.0104	
Fish	0.260	0.107	0.0181	
Cured meat	0.259	0.121	0.0361	
Coffee	0.217	0.108	0.0488	
Sweetbread	0.212	0.117	0.0768	
Raw vegetables	0.145	0.102	0.1624	
Butter	−0.386	0.104	0.0005	
Poultry	−0.226	0.105	0.0359	
Potatoes	−0.189	0.115	0.1066	
Jam	−0.148	0.117	0.2097	
Cu/Zn (copper/zinc molar ratio)				
Milk	0.351	0.125	0.0067	0.17
Butter	0.172	0.127	0.1814	
Sugar	0.137	0.118	0.2506	
Oils	−0.246	0.120	0.0444	
Margarine	−0.251	0.126	0.0503	
Honey	−0.179	0.125	0.1571	
Cottage cheese	−0.136	0.119	0.2573	
TAS (total antioxidant status)				
Tea	0.418	0.115	0.0006	0.25
Coffee	0.181	0.112	0.1109	
Sweetbread	0.183	0.119	0.1304	
Milk	0.172	0.116	0.1446	
Whole-grain bread	0.141	0.117	0.2360	
Full-fat cheese	0.127	0.111	0.2599	
Groats and rice	−0.267	0.122	0.0327	
Sausages	−0.215	0.115	0.0680	
Jam	−0.173	0.118	0.1468	
Cottage cheese	−0.198	0.123	0.1123	
Raw vegetables	−0.154	0.114	0.1814	
TOS (total oxidant status)				
Margarine	0.406	0.118	0.0012	0.33
Fish	0.242	0.115	0.0397	
White bread	0.260	0.134	0.0577	
Groats and rice	0.194	0.118	0.1068	
Butter	0.144	0.120	0.2357	
Meat	0.133	0.115	0.2506	
Milk	−0.323	0.115	0.0068	
Tea	−0.315	0.115	0.0082	
Boiled vegetables	−0.272	0.118	0.0256	
Potatoes	−0.255	0.121	0.0397	
Coffee	−0.196	0.105	0.0680	
Sugar	−0.195	0.124	0.1234	
Full-fat cheese	−0.181	0.116	0.1246	
Farinaceous dishes	−0.136	0.116	0.2463	
OSI (oxidative stress index)				
Groats and rice	0.260	0.123	0.0403	0.33
Margarine	0.226	0.110	0.0455	
Honey	0.197	0.119	0.1019	
White bread	0.203	0.122	0.1033	
Sausages	0.151	0.116	0.1985	
Poultry	0.141	0.113	0.2166	
Tea	−0.404	0.111	0.0006	
Milk	−0.315	0.117	0.0097	
Boiled vegetables	−0.301	0.118	0.0137	
Potatoes	−0.291	0.121	0.0190	
Coffee	−0.242	0.106	0.0270	
Full-fat cheese	−0.199	0.116	0.0921	
Sugar	−0.163	0.119	0.1758	

Statistically significant differences between the levels were detected using stepwise multiple linear regression analysis. Abbreviations: SE—standard error, *p*—level of significance.

**Table 6:** Dietary intake of nutrients and the percentage of ARC patients whose diets complied with nutritional standards.

Median (Q1–Q3)		ARC Group			*p*-Value F vs. M
		F (*n* = 43)	M (*n* = 25)	All (*n* = 68)	
Na (sodium)					
Norm: AI_F_ = 1500 mg/day; AI_M_ = 1500 mg/day					
3269.788 (2637.895–4008.582)	Below: *n* (%)	2 (5%)	0 (0%)	2 (3%)	<0.005
	Above: *n* (%)	41 (95%)	25 (100%)	66 (97%)	
K (potassium)					
Norm: AI_F_ = 3500 mg/day; AI_M_ = 3500 mg/day					
3044.111 (2531.669–4001.523)	Below: *n* (%)	31 (72%)	17 (68%)	48 (71%)	ns
	Above: *n* (%)	12 (28%)	8 (32%)	20 (29%)	
Ca (calcium)					
Norm*: EAR_F_ = 800/1000 mg/day; EAR_M_ = 800/1000 mg/day					
496.749 (314.227–756.352)	Below: *n* (%)	41 (95%)	20 (80%)	61 (90%)	ns
	Above: *n* (%)	2 (5%)	5 (20%)	7 (10%)	
P (phosphorus)					
Norm: EAR_F_ = 580 mg/day; EAR_M_ = 580 mg/day					
1130.194 (855.115–1406.868)	Below: *n* (%)	1 (2%)	0 (0%)	1 (1.5%)	ns
	Above: *n* (%)	42 (98%)	25 (100%)	67 (98.5%)	
Mg (magnesium)					
Norm: EAR_F_ = 265 mg/day; EAR = 350 mg/day					
271.096 (219.470–347.230)	Below: *n* (%)	23 (53%)	18 (72%)	41 (60%)	ns
	Above: *n* (%)	20 (47%)	7 (28%)	27 (40%)	
Fe (iron)					
Norm: EAR_F_ = 6 mg/day; EAR_M_ = 6 mg/day					
9.768 (7.180–12.582)	Below: *n* (%)	6 (14%)	1 (4%)	7 (10%)	ns
	Above: *n* (%)	37 (86%)	24 (96%)	61 (90%)	
Zn (zinc)					
Norm: EAR_F_ = 6.8 mg; EAR_M_ = 9.4 mg					
8.652 (6.614–11.978)	Below: *n* (%)	15 (35%)	9 (35%)	24 (35%)	ns
	Above: *n* (%)	28 (65%)	16 (65%)	44 (65%)	
Cu (copper)					
Norm: EAR_F_ = 0.7 mg/day; EAR_M_ = 0.7 mg/day					
1.059 (0.867–1.396)	Below: *n* (%)	9 (21%)	2 (8%)	11 (16%)	ns
	Above: *n* (%)	34 (79%)	23 (92%)	57 (84%)	
Mn (manganese)					
Norm: AI_F_ = 1.8 mg/day; AI_M_ = 2.3 mg/day					
3.671 (2.461–5.569)	Below: *n* (%)	6 (14%)	4 (16%)	10 (15%)	ns
	Above: *n* (%)	37 (86%)	21 (84%)	58 (85%)	
I (iodine)					
Norm: EAR_F_ = 95 µg/day; EAR_M_ = 95 µg/day					
141.309 (96.942–170.633)	Below: *n* (%)	11 (26%)	4 (16%)	15 (22%)	ns
	Above: *n* (%)	32 (74%)	21 (84%)	53 (78%)	
Vitamin A (retinol)					
Norm: EAR_F_ = 500 µg/day; EAR_M_ = 630 µg/day					
954.291 (665.553–1219.684)	Below: *n* (%)	6 (14%)	4 (16%)	10 (15%)	<0.05
	Above: *n* (%)	37 (86%)	21 (84%)	58 (85%)	
Vitamin B_1 (_thiamine)					
Norm: EAR_F_ = 0.9 mg/day; EAR_M_ = 1.1 mg/day					
1.131 (0.837–1.472)	Below: *n* (%)	18 (42%)	6 (24%)	24 (35%)	<0.005
	Above: *n* (%)	25 (58%)	19 (76%)	44 (65%)	
Vitamin B_2_ (riboflavin)					
Norm: EAR_F_ = 0.9 mg/day; EAR_M_ = 1.1 mg/day					
1.414 (1.055–1.887)	Below: *n* (%)	4 (9%)	5 (20%)	9 (13%)	ns
	Above: *n* (%)	39 (81%)	20 (80%)	59 (87%)	
Vitamin B_3_ (niacin)					
Norm: EAR_F_ = 11 mg/day; EAR_M_ = 12 mg/day					
15.985 (12.799–20.362)	Below: *n* (%)	5 (12%)	5 (20%)	10 (15%)	ns
	Above: *n* (%)	38 (88%)	20 (80%)	58 (85%)	
Vitamin B_6_ (pyridoxine)					
Norm**: EAR_F_ = 1.1/1.3 mg/day; EAR_M_ = 1.1/1.4 mg/day					
1.886 (1.512–2.180)	Below: *n* (%)	9 (26%)	4 (16%)	13 (19%)	ns
	Above: *n* (%)	34 (74%)	21 (84%)	55 (81%)	
Vitamin B_9_ (folic acid)					
Norm: EAR_F_ = 400 µg/day; EAR_M_ = 100 µg/day					
240.445 (173.437–312.437)	Below: *n* (%)	41 (95%)	20 (80%)	61 (90%)	ns
	Above: *n* (%)	2 (5%)	5 (20%)	7 (10%)	
Vitamin B_12_ (cyanocobalamin)					
Norm: EAR_F_ = 2 µg/day; EAR_M_ = 2 µg/day					
2.418 (1.710–4.046)	Below: *n* (%)	19 (44%)	8 (32%)	27 (40%)	ns
	Above: *n* (%)	24 (56%)	17 (68%)	41 (60%)	
Vitamin C (Ascorbic acid)					
Norm: EAR_F_ = 60 mg/day; EAR_M_ = 75 mg/day					
83.175 (39.130; 146.091)	Below: *n* (%)	18 (42%)	12 (48%)	30 (44%)	ns
	Above: *n* (%)	25 (58%)	13 (52%)	38 (56%)	
Vitamin D (calciferol)					
Norm: AI_F_ = 15 µg/day; AI_M_ = 15 µg/day					
1.941 (1.353–2.995)	Below: *n* (%)	43 (100%)	25 (100%)	68 (100%)	ns
	Above: *n* (%)	0 (0%)	0 (0%)	0 (0%)	
Vitamin E (tocopherol)					
Norm: AI_F_ = 8 mg/day; AI_M_ = 10 mg/day					
9.268 (7.191–12.765)	Below: *n* (%)	15 (35%)	11 (44%)	26 (38%)	ns
	Above: *n* (%)	28 (65%)	14 (56%)	42 (62%)	
DHA+EPA					
Norm: AI_F_ = 250 mg/day; AI_M_ = 250 mg/day					
58.633 (36.117–112.517)	Below: *n* (%)	37 (86%)	19 (76%)	56 (82%)	ns
	Above: *n* (%)	6 (14%)	6 (24%)	12 (18%)	
NaCl (sodium chloride)					
Norm: 5 g/day					
8.179 (6.599–9.851)	Below: *n* (%)	4 (9%)	0 (0%)	4 (6%)	<0.005
	Above: *n* (%)	39 (91%)	25 (100%)	64 (94%)	
Protein					
Norm: EAR_F_ = 0.66 g/kg BW; EAR_M_ = 0.66 g/kg BW					
1.003 (0.823–1.222)	Below: *n* (%)	4 (9%)	1 (4%)	5 (7%)	ns
	Above: *n* (%)	39 (91%)	24 (96%)	63 (93%)	
Fats					
RI: 30–40% E ^1^					
33.255 (30.775–36.825)	Below: *n* (%)	12 (28%)	2 (8%)	14 (21%)	<0.05
	Above: *n* (%)	31 (72%)	23 (92%)	54 (79%)	
Carbohydrates					
RI: 45–65% E ^1^					
46.650 (43.525–50.125)	Below: *n* (%)	11 (26%)	12 (48%)	23 (34%)	<0.05
	Above: *n* (%)	32 (74%)	13 (52%)	45 (66%)	
Fiber					
Norm***: AI_F_ = 25/20 mg; AI_M_ = 25/20 mg					
19.142 (14.506–28.285)	Below: *n* (%)	27 (63%)	11 (26%)	38 (56%)	ns
	Above: *n* (%)	16 (37%)	14 (74%)	30 (44%)	

Statistically significant differences between the medians were detected using the Mann–Whitney U test. Abbreviations: ARC—age-related cataract, F—female, M—male, *p*—level of significance, ns—not significant, EAR—estimated average requirement, AI—adequate intake, RI—reference intake ranges for macronutrients, BW—body weight. Norm* of EAR for Ca: for women aged 31–50 = 800 mg; for women aged 51 and over = 1000 mg; for men aged 31–65 = 800 mg; for men aged 66 and over = 1000 mg. Norm** of EAR for vitamin B_6_: for women aged 31–50 = 1.1 mg; for women aged 51 and over = 1.3 mg; for men aged 31–65 = 1.1 mg; for men aged 66 and over = 1.4 mg. Norm*** of EAR for fiber: for women aged 31–65 = 25 mg; for women aged 66 and over = 20 mg; for men aged 31–65 = 25 mg; for men aged 66 and over = 20 mg; EPA—eicosapentaenoic acid, DHA—docosahexaenoic acid, ^1^—% of total energy that comes from the macronutrient.

**Table 7:** Correlations between blood serum parameters and dietary intake of nutrients or lifestyle choices among patients with ARC.

Variable 1	Variable 2	R	*p*-Value
TOS	Dietary vitamin A	−0.25	<0.05
	Dietary β-carotene	−0.26	<0.05
Serum Zn	Smoking status	−0.24	<0.05
Cu/Zn molar ratio	Dietary Zn	−0.35	<0.01
	Dietary Cu	−0.28	<0.05
	Dietary Mn	−0.29	<0.05

Statistically significant correlations were detected using Spearman’s correlation coefficient. Abbreviations: TOS—total oxidant status, Cu/Zn molar ratio—copper/zinc molar ratio, Zn—zinc, Cu—copper, Mn—manganese, *p*—level of significance.

## Data Availability

The raw data supporting the conclusions of this article are included in the Supplementary Materials. Further inquiries can be directed to the corresponding author.

## Supplementary Materials

The following supporting information can be downloaded at https://www.mdpi.com/article/10.3390/nu17203237/s1. Material S1: Raw data supporting the conclusion of the article; Figure S1. Scatterplot of vitamin A [µg/day] against TOS [µmol H2O2 equiv./L]; Figure S2. Scatterplot of β-carotene [µg/day] against TOS [µmol H2O2 equiv./L]; Figure S3. Scatterplot of smoking status against serum Zn [mg/L] levels; Figure S4. Scatterplot of dietary Zn intake [mg/day] against Cu/Zn molar ratio; Figure S5. Scatterplot of dietary Cu intake [mg/day] against Cu/Zn molar ratio; Figure S6. Scatterplot of dietary Mn intake [mg/day] against Cu/Zn molar ratio.

## Abbreviations

The following abbreviations are used in this manuscript:

AAS	Atomic absorption spectrometry
ARC	Age-related cataract
ATP	Adenosine triphosphate
BMI	Body mass index
BP	Blood pressure
CAT	Catalase
Cu	Copper
Cu/Zn molar ratio	Copper to Zn molar ratio
DHA	Docosahexaenoic acid
DNA	Deoxyribonucleic acid
ECG	Electrocardiography
EPA	Eicosapentaenoic acid
EPIC-Oxford	European Prospective Investigation into Cancer and Nutrition
FBS	Fasting blood sugar
FFQ	Food frequency questionnaire
GPX	Glutathione peroxidase
GSH	Glutathione
HR	Heart rate
LFTs	Liver function tests
LOCS III	Lens Opacities Classification System III
M	Mean
Me	Median
Mn	Manganese
MSVI	Moderate to severe vision impairment
mtDNA	Mitochondrial DNA
MUB	Medical University of Białystok
NO	Nitric oxide
OR	Odds Ratio
OS	Oxidative stress
OSI	Oxidative stress index
PRS	Peripheral blood smear
PSC	Posterior subcapsular cataract
ROS	Reactive oxygen species
Se	Selenium
SOD	Superoxide dismutase
TAC	Total antioxidant capacity
TAS	Total antioxidant status
TOS	Total oxidant status
TrxR	Thioredoxin reductase
UV	Ultraviolet
Zn	Zinc
