# Supplementary material for: Influence of Dietary Habits on Oxidative Stress Parameters, Selenium, Copper, and Zinc Levels in the Serum of Patients with Age-Related Cataract
Source: Nutrients. 2025 Oct 15;17(20):3237. doi: 10.3390/nu17203237 (PMC12566761; doi:10.3390/nu17203237)
Supplement: Supplementary file 1 [file nutrients-17-03237-s001.zip › Supplementary figures (nutrients-3906836).pdf]

Supplementary Material

Influence of Dietary Habits on Oxidative Stress Parameters, Selenium, Copper, and Zinc Levels in the Serum of Patients with Age-Related Cataract

Martyna Falkowska [1,*], Izabela Zawadzka [2], Monika Grabia-Lis [1], Dominika Patrycja Dobiecka [1], Maryla Młynarczyk [2], Joanna Konopińska [2], and Katarzyna Socha [1]

[1] Department of Bromatology, Faculty of Pharmacy with the Division of Laboratory Medicine, Medical University of Białystok, Mickiewicza 2D Street, 15-222 Białystok, Poland; monika.grabia@umb.edu.pl, dominika.dobiecka@sd.umb.edu.pl, katarzyna.socha@umb.edu.pl

[2] Department of Ophthalmology, Medical University of Białystok, M. Skłodowskiej-Curie 24a, 15-276 Białystok, Poland; cwalinaizabela@gmail.com, mromaniuk2121@gmail.com, joannakonopinska@o2.pl

* Correspondence: martyna.falkowskaa@gmail.com

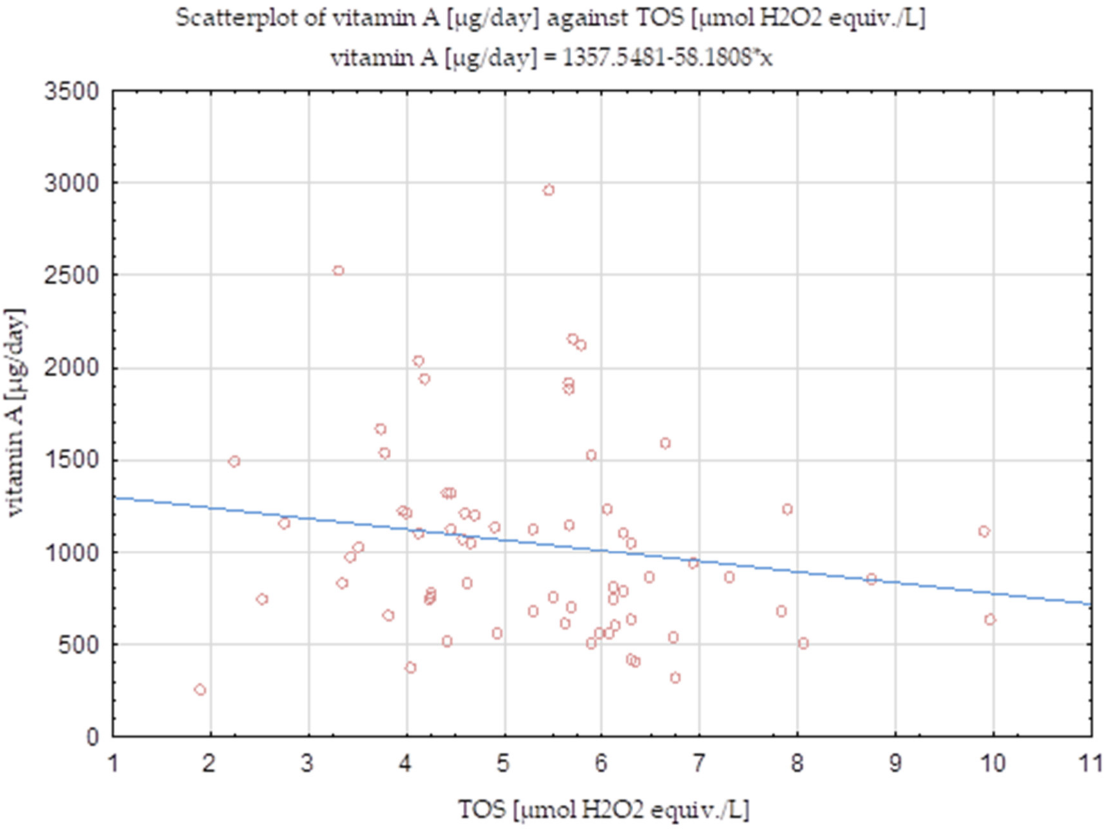

Figure S1. Scatterplot of vitamin A [µg/day] against TOS [µmol H2O2 equiv./L]

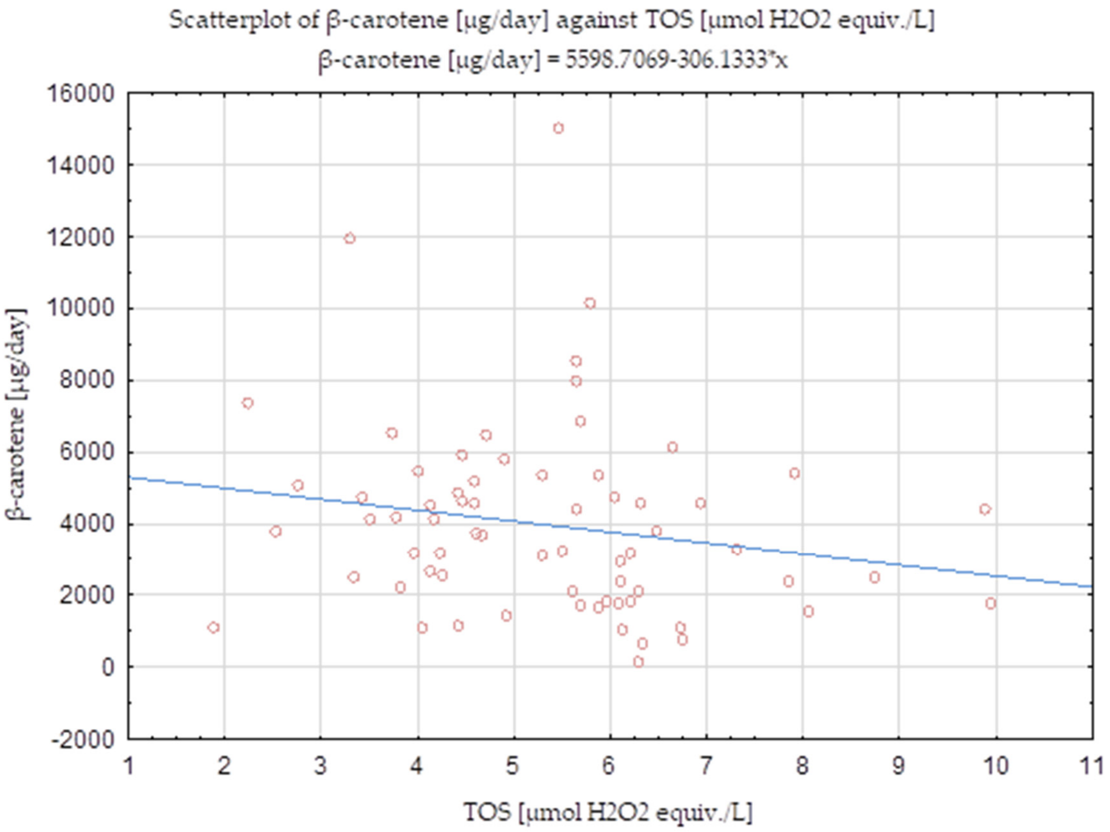

Figure S2. Scatterplot of β-carotene [µg/day] against TOS [µmol H2O2 equiv./L]

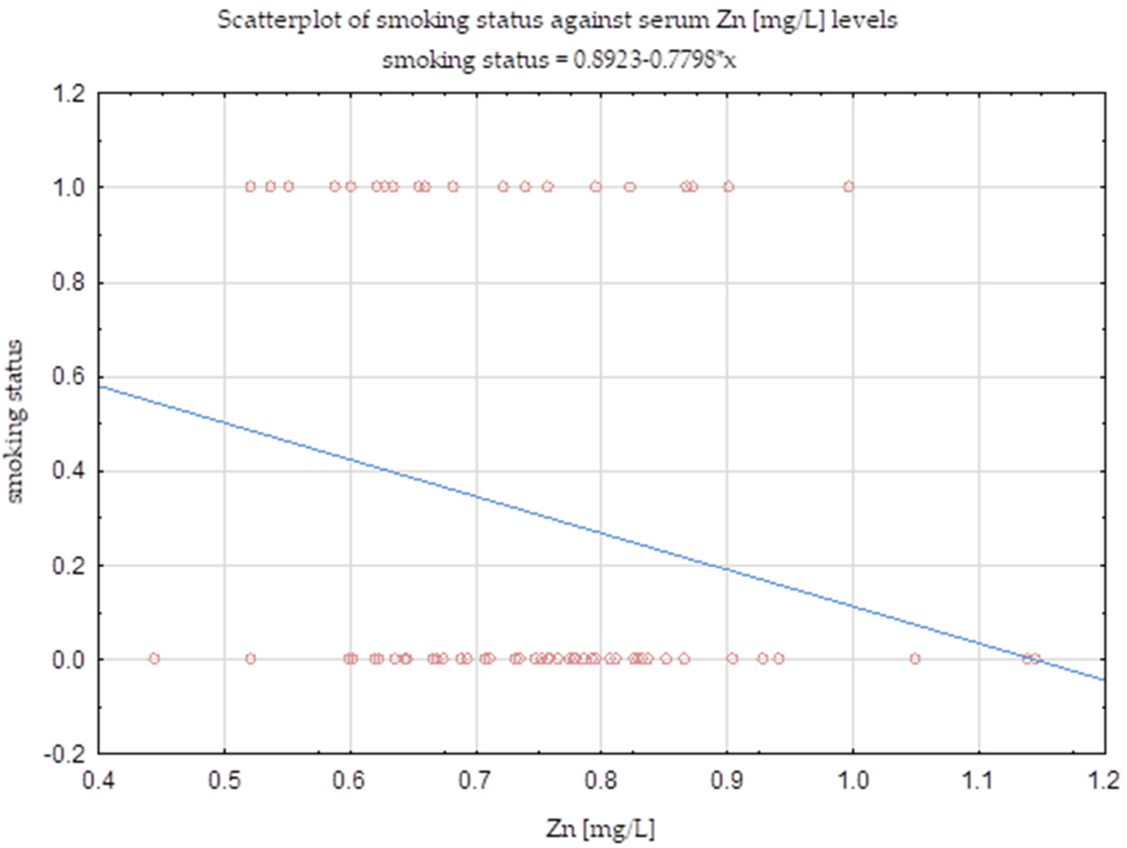

Figure S3. Scatterplot of smoking status against serum Zn [mg/L] levels

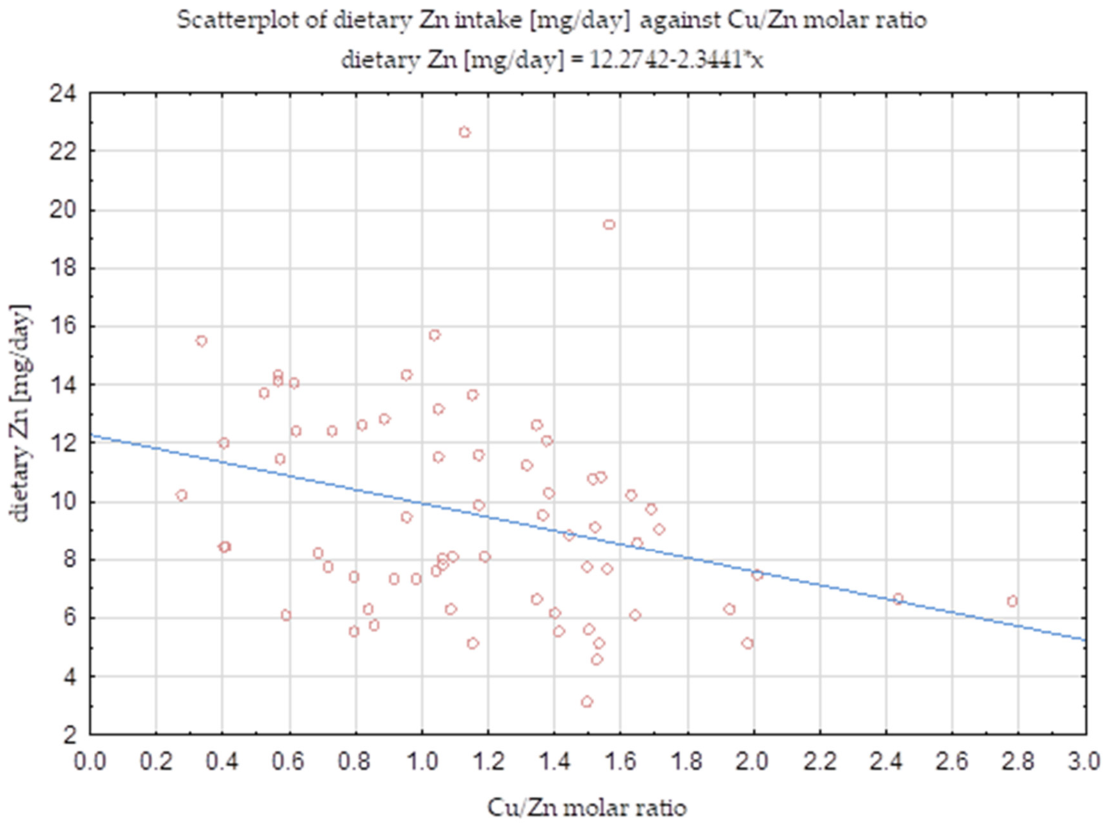

Figure S4. Scatterplot of dietary Zn intake [mg/day] against Cu/Zn molar ratio

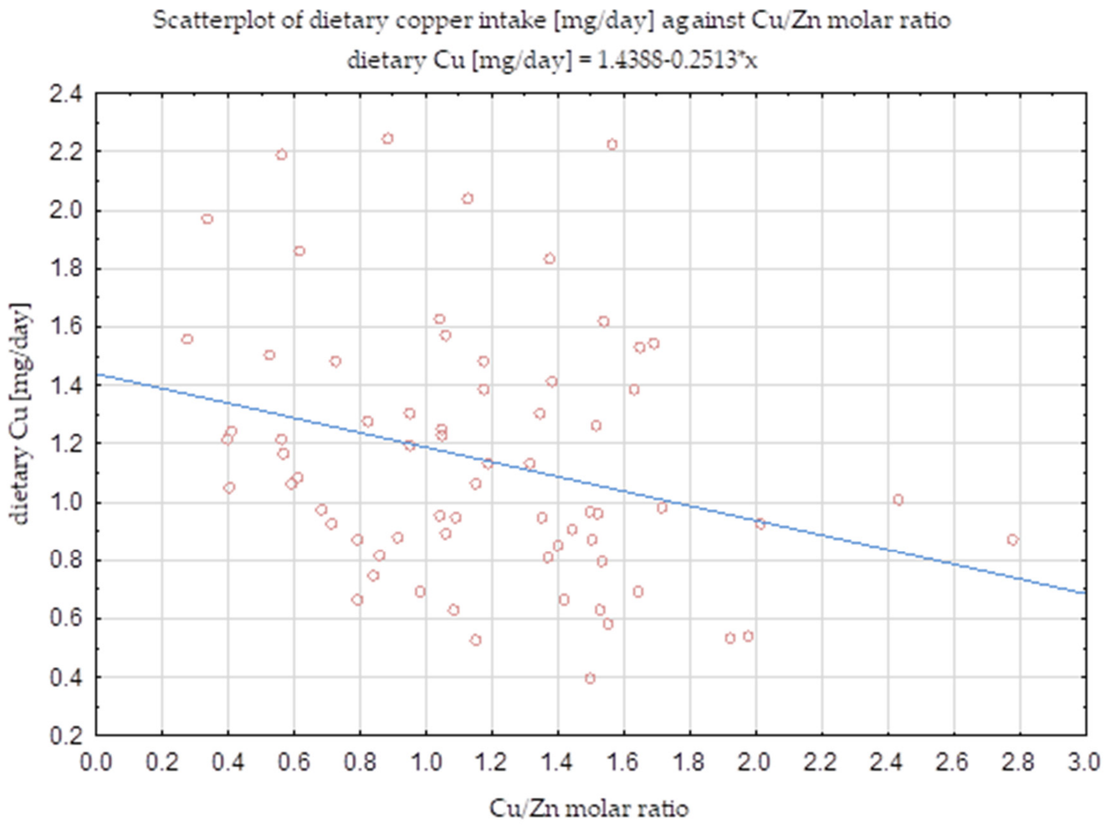

Figure S5. Scatterplot of dietary Cu intake [mg/day] against Cu/Zn molar ratio

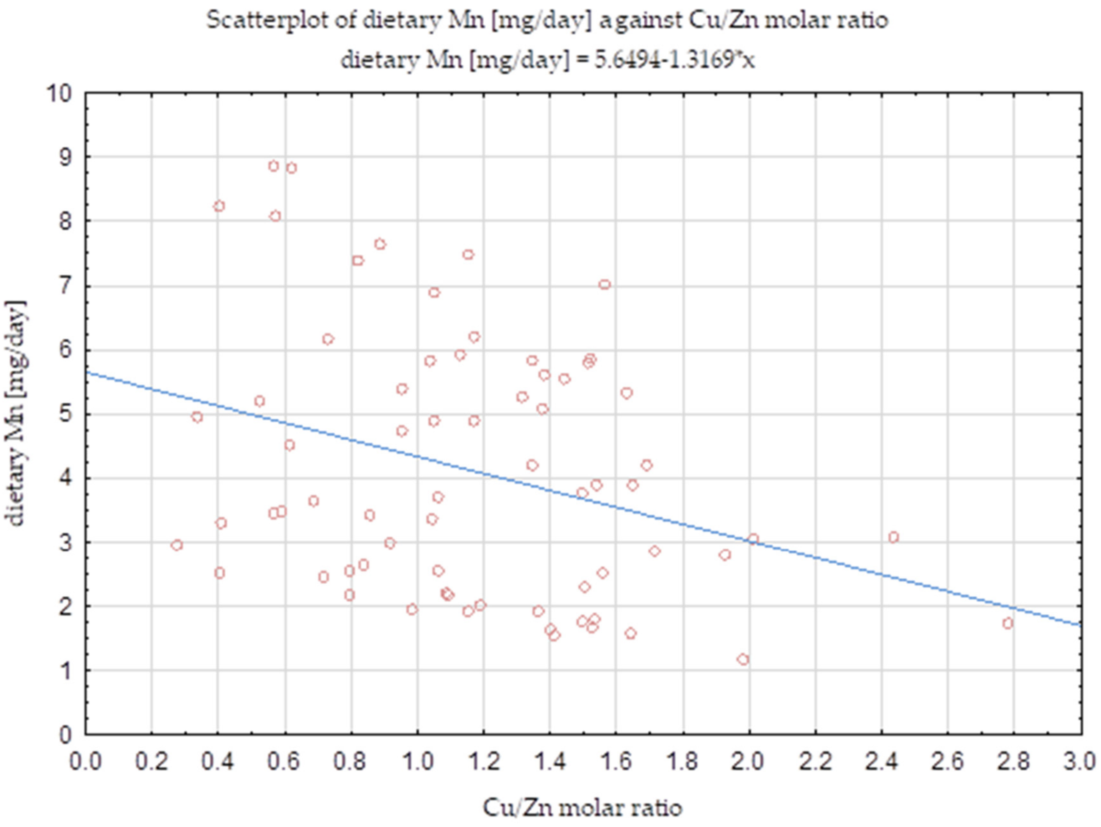

Figure S6. Scatterplot of dietary Mn intake [mg/day] against Cu/Zn molar ratio
